# The Threat of Instability: Neurodegeneration Predicted by Protein Destabilization and Aggregation Propensity

**DOI:** 10.1371/journal.pbio.0060193

**Published:** 2008-07-29

**Authors:** Elizabeth M Meiering

## Abstract

A new method based on protein stability and aggregation propensity reveals a strong correlation between the properties of mutant Cu/Zn-superoxide dismutases associated with amyotrophic lateral sclerosis and patient survival.

In 1961, elegant experiments by Christian Anfinsen first demonstrated that a protein, the enzyme ribonuclease A, could be reversibly denatured (unfolded) and subsequently renatured (refolded) to its functional native state [1]. This and subsequent similar findings for many other proteins gave rise to a central tenet of protein folding, that the information specifying how a protein attains its native state is encoded in its primary amino acid sequence. Thus was posed “The Protein Folding Problem,” which remains a central unsolved problem in biology: exactly how does a protein's primary sequence determine its structure and function, or its misfolding and misfunction?

Until the late 1980s, protein folding was studied by relatively few scientists, and the problem was generally regarded as fundamental, but of minimal immediate, practical importance. With the advent of massive genome sequencing efforts and the concomitant recognition that many diseases are caused by single amino acid substitutions in many different proteins, the drive to understand how primary sequence governs folding and function greatly intensified. A common feature of numerous mutation-linked diseases is the deposition of misfolded protein aggregates. Extensive studies have shown that protein aggregates are often misfunctional, i.e., toxic to cells, but the nature and targets of the toxic aggregated species are not well understood and are still under intense investigation. Anfinsen's and related experiments also established that the folding of an unfolded protein is not trivial, depending strongly on solution conditions, and protein misfolding and aggregation reactions typically compete with folding reactions. Furthermore, the difference in energy between the native folded state and the unfolded state of a protein, which defines its thermodynamic stability, is typically small (20–40 kJ mol^−1^), corresponding to the energy of one to several noncovalent interactions (such as a hydrogen bond, or salt bridge) among the myriad of such interactions occurring in both the folded and unfolded states [2]. As a consequence, it is quite easy to significantly alter the relative proportions of protein occupying the folded versus fully or partially unfolded states, for example by substitution of a single amino acid, or by a covalent modification such as proteolysis (as is also frequently observed for proteins associated with misfolding diseases).

Thus, it appears that many proteins have evolved to be only as stable as they need to be in order to remain largely folded and active, but also sufficiently unstable to be easily turned over or regulated. Some proteins are not even folded into a defined globular shape, and instead populate a fluctuating, natively unfolded state that can switch to a folded state upon binding of a suitable partner molecule. Such natively unfolded proteins have different characteristics from native globular proteins, such as a higher proportion of charged amino acids and lower proportion of hydrophobic amino acids, which decrease their tendency to aggregate [3].

## Molecular Details and Consequences of Protein Aggregation

The molecular details of protein aggregation are in general not well understood, owing to the complexity and variability of misfolding reactions and technical difficulties in characterizing aggregates, due to their often heterogeneous and fibrillar nature. Aggregate structures can range from poorly ordered (amorphous) to highly ordered, and the same protein can adopt a range of structures, depending on solution conditions. Both amorphous and ordered aggregates are often enriched in beta-sheet secondary structure. A common type of beta-sheet-based, ordered aggregate structure that has been studied extensively is known as amyloid [4]. It has been proposed that the ability to form amyloid structure is an inherent or generic property of polypeptide chains, although the propensity to do so can vary dramatically with primary sequence. Amyloid formation is associated with a host of diseases—notably prevalent neurodegenerative diseases, including Alzheimer, Parkinson, and Huntington diseases as well as prion diseases, but also non-neuropathic systemic and localized diseases. Amyloid aggregates consist of long unbranched fibers of a given protein, which forms a “cross-beta” structure in which beta-strands are oriented perpendicular to the axis of the fiber. Also, they exhibit “apple-green” birefringence upon staining with Congo red, a dye long used by pathologists to diagnose misfolding diseases. The amyloidogenic proteins in the different diseases are highly diverse in terms of their primary sequences, native structures, and functions, and include both folded globular and natively unfolded proteins. Aggregate structures associated with other diseases do not fit all the criteria for amyloid, but are often also fibrillar [4,5].

In this issue of *PLoS Biology*, Wang et al. [6] investigate the basis for the formation of nonamyloid, fibrillar aggregates by Cu/Zn-superoxide dismutase (SOD1) in another neurodegenerative disease: amyotrophic lateral sclerosis (ALS), also known as Lou Gehrig disease. ALS is an invariably fatal, rapidly progressive motor neuron disease for which there is no cure and very little in the way of effective treatment. In general, treatments for neurodegenerative diseases are very limited, which imparts great urgency to research aimed at understanding and combating these devastating and widespread diseases.

## Principles and Prediction of Protein Aggregation and Relation to Disease

What common principles may underlie the aggregation of so many different proteins in many different diseases? Aggregation is thought usually to occur from partially to fully unfolded states of proteins. It is well established that destabilization of the native state plays an important role in the pathogenic effects of mutations of various globular proteins [7]. Examples include mutations of lysozyme [8,9], transthyretin [10], and immunoglobulin light chains [11,12] in various amyloidoses, and serpins that form various nonamyloid aggregates in serpinopathies [5]. Seminal research by Dobson and colleagues established an equation (referred to here as the Chiti-Dobson equation) for predicting the aggregation propensity of unfolded proteins based on their biophysical properties [13]. This equation has been applied extensively to amyloid formation by mutant proteins or peptides associated with disease or toxicity. In the Chiti-Dobson equation, the calculated aggregation propensity of a protein is increased when mutations decrease net charge, increase hydrophobicity, increase beta-sheet propensity, or decrease alpha-helix propensity.

Wang et al. have advanced research on protein folding and aggregation by building on existing knowledge in two important ways: (1) applying the Chiti-Dobson equation to calculate aggregation propensity for mutant forms of SOD1 associated with ALS, a nonamyloid disease; and (2) combining calculated aggregation propensity quantitatively with measured experimental stability of mutant SOD1 to define a strong correlation with ALS disease severity as measured by patient survival time (i.e., disease duration) [6]. In contrast with some other diseases, however, no trends were identified between the nature of SOD1 mutations and age of disease onset. SOD1 is a homodimeric metalloenzyme, with each subunit consisting of a 153 amino acid chain that binds a structural zinc ion and a catalytic copper ion. Over 110, predominantly missense, mutations located throughout the SOD1 structure have been linked to familial forms of ALS (http://alsod.iop.kcl.ac.uk/ALS/index.aspx). It has been proposed previously that decreased stability of unmetallated (apo) SOD1 is correlated with decreased disease duration [14], and that disease-associated SOD1 mutations have a tendency to decrease protein net charge and hence favor aggregation [15,16]; however, these trends have been questioned [6,15]. Key problems with identifying reliable trends have included limited patient data, as well as a lack of systematic, accurate analyses of sufficient numbers of mutant SOD1s.

**Figure 1:**
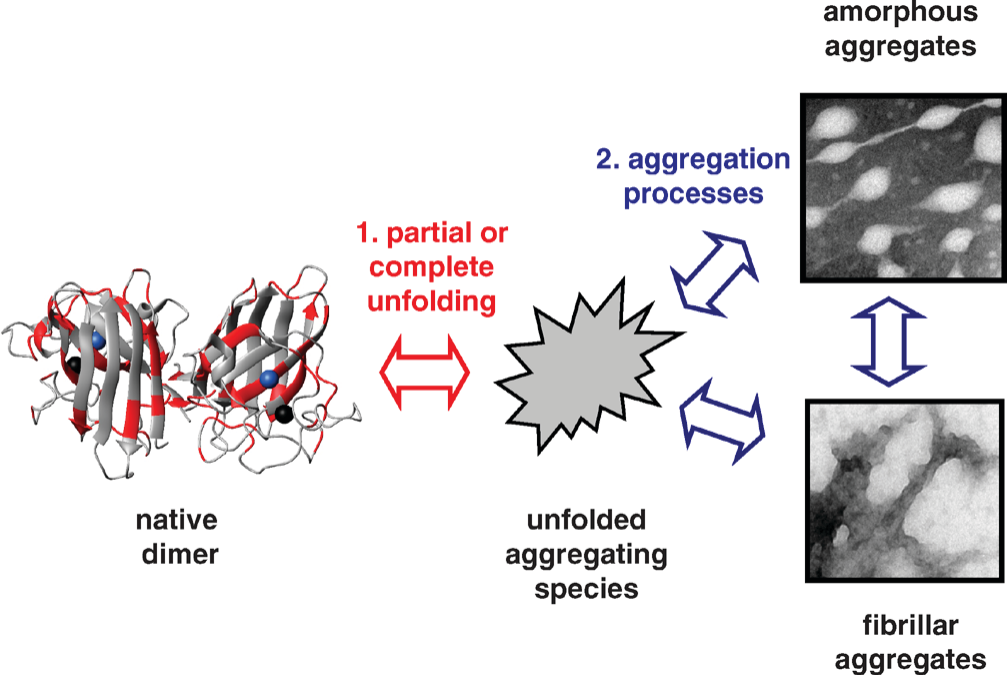
Misfolding and Aggregation of SOD1 Wang et al.'s correlation of mutant SOD1 properties with disease duration [6] implicates two steps in the formation of aggregates of SOD1 in ALS: (1) native homodimeric metalloprotein, shown in ribbon representation (left) partially or completely unfolds to form a range of possible dimeric or monomeric aggregation-prone species, shown as a general grey irregular shape (middle), followed by (2) progressive assembly of the aggregation-prone species to form initially small soluble and later fibrillar aggregates (right panels). The structure of native SOD1 was generated using MolMol [29] and Protein Data Bank accession code 1n18. Sites of ALS-associated mutations studied by Wang et al. are shown in red; bound copper and zinc ions are shown as blue and black spheres, respectively. The right panels are transmission electron microscopy images of apo SOD1 aggregates formed in vitro that strongly resemble granular and granule-coated fibrillar SOD1 aggregates observed in ALS [19].

Wang et al. collected from the literature the most extensive sets of patient data and biophysical measurements to date. They applied a recalibrated Chiti-Dobson equation to ALS mutant SOD1 and found that increased aggregation propensity is significantly correlated with decreased patient survival. They also further validated that decreased mutant SOD1 stability tends to be associated with decreased survival. By then combining the effects of mutations on both aggregation propensity and stability, they found that the correlation with survival increases considerably, accounting for 69% of the variability in the ALS patient survival data. The improved correlation supports an intuitive and attractive physical model in which ALS is promoted by a decrease in SOD1 stability, which favors partial or complete unfolding, and this in turn favors formation of toxic aggregates (Figure 1); this type of model is likely to be applicable to many other misfolding disease scenarios.

## Challenges and Approaches to Unraveling Protein Aggregation

A key question now is: why does the Wang et al. analysis not account for 100% of the SOD1 mutant patient data? There are many possible contributing factors. Foremost among these are serious limitations in the amount and quality of available patient data. Another important factor is limitations in protein stability data. Wang et al. have used a reasonable, but unorthodox, normalization approach to rank relative stabilities of mutant proteins by combining measurements of stability and apparent melting temperatures for apo SOD1, acquired under different conditions and with different methodologies. This can contribute to variability in a number of ways, which are likely offset to some extent by the averaging procedure employed by the authors. A key point for future studies, however, is to bear in mind possible systematic lowering of apparent melting temperatures if thermal unfolding is accompanied by aggregation, as could frequently occur [17,18]. This will likely affect apo and especially metallated SOD1 stabilities to varying extents for different states of different mutants. Also, the use of apo SOD1 stability, which is relatively lower and so can favor aggregation compared to metallated protein [19,20], may not report on all relevant aggregation processes. There is also evidence for aggregation of full- or part-metallated states of SOD1 [21,22], as well as aggregation of SOD1s altered by covalent modification such as oxidation or proteolysis [23,24]. Considering all these data, it appears that SOD1 may be poised to aggregate, likely in part due to its high concentration in neural cells, via a range of pathways.

There are still more complexities that may weaken the correlation for the SOD1 patient data analysis. Wang et al. find that increased SOD1 net charge is weakly correlated with decreased survival time, opposite to the trends reported by Chiti and Dobson [13] and by other studies of SOD1 mutations [15,16]. This requires further investigation, and may give clues to the perhaps oppositely charged targets of SOD1 and other protein aggregates. Finally, although different SOD1 mutations tend to be associated with different average disease durations, the actual durations for individuals with a given mutation are highly variable [6,25,26,27]. Such variability is not a special case, but is observed for many diseases. For ALS and other neurodegenerative diseases, it is increasingly apparent that variability occurs because additional effects, including age-dependent, environmental, and other genetic factors, modulate disease [27,28]. Further analysis of these factors will be critical for reaching a deep understanding of these complex diseases; an essential component will be the collection and reporting of comprehensive patient data by physicians.

Clearly much remains to be learned about protein misfolding and aggregation in disease. The Wang et al. study has moved the field forward by formulating a quantitative analysis that better accounts for patient data by incorporating multiple factors to describe the complex effects of SOD1 mutations. This provides a useful framework for developing further improved predictive equations by including additional effects of disease modulators, testing disease predictions, and pursuing common and distinct targets of protein aggregates in order to develop urgently needed therapeutic approaches.

## Abbreviations

ALS: amyotrophic lateral sclerosis
SOD1: Cu/Zn-superoxide dismutase
